# Factors affecting the safe sexual behaviors of Korean young adults by gender: a structural equation model

**DOI:** 10.4069/kjwhn.2023.06.16

**Published:** 2023-06-30

**Authors:** Nalae Moon, Hyunjin Kang, Su Ji Heo, Ju Hee Kim

**Affiliations:** College of Nursing Science, Kyung Hee University, Seoul, Korea

**Keywords:** Attitude, Communication, Safe sex, Sex characteristics, Theory of planned behavior

## Abstract

**Purpose:**

The aim of this study was to determine the factors that influence safe sexual behaviors of Korean young adults and identify differences by gender.

**Methods:**

This study aimed to determine which factors affected safe sexual behaviors based on the Theory of Planned Behavior. Data from 437 Korean young adults (in their 20s and 30s) were collected via online survey between January 3 and January 28, 2022. The questionnaire included items on sexual body image, sexual role perception, sexual attitudes, sexual socialization, sexual communication, and safe sexual behaviors. Structural equation modeling was performed.

**Results:**

According to the overall model fit of the hypothetical model, the final model was acceptable and explained 49% of safe sexual behaviors. Sexual attitudes (β=–.70, *p*<.001) and sexual communication (β=.53, *p*<.001) directly affected safe sexual behaviors, and sexual role perception (β=.42, *p*<.001) indirectly affected safe sexual behaviors in a combined model. There were gender differences in the path from sexual attitudes (β=–.94, *p*<.001) and sexual communication (β=.66, *p*<.001) to safe sexual behaviors and from sexual body image (β=.27, *p*<.001) to sexual communication.

**Conclusion:**

Sexual attitudes and sexual communication were predictors of safe sexual behaviors, which differed by gender. Strategies that consider sexual attitudes, sexual communication, sexual role perception, and differences between men and women should be developed to improve the safe sexual behaviors of young adults.

## Introduction

According to the World Health Organization and the Centers for Disease Control and Prevention news update in 2022 [1], more than 1 million people worldwide are newly diagnosed with sexually transmitted infections each day, with 45%, 52%, and 235% increases in gonorrhea (n=677,769), syphilis (n=133,945), and congenital syphilis (n=2,148) cases, respectively, since the 2016 report [2]. Despite various campaigns and educational programs, condom use, which is the most effective way to prevent sexually transmitted infections, has not increased significantly over the past decade and awareness of condom use among young adults is lower than that among those in other age groups [2,3]. A study from the United States that analyzed trends in condom use from 2002 to 2017 identified condom use rates of 64.4% in 2002, 70.0% from 2006 to 2010, 65.6% from 2011 to 2015, and 67.1% from 2015 to 2017 in women aged 20 to 24 years old.

According to a recent national report in South Korea (hereafter Korea), there are changing trends in family structure among young adults aged 20 to 30 years old. The proportion of single-person households in Korea increased by more than 3.5 times in 2020 (31.7%) since 1990 (9.0%) [4]. Single-person households made up 74.8% of the households of those in their 20s and 35.7% of the households of those in their 30s, compared to the 20% proportion of single-person households among those in their 40s and 50s [4]. In 2021, there were 192,507 marriages in Korea, indicating a decrease of about 10% compared to 2020. The number of marriages has steadily declined since 2011, falling below 200,000 for the first time in 2021.

While the legal marriage rate is falling, the perception of cohabitation and free sexual relationships among young adults is becoming more acceptable, with 68% of men and 62% of women in Korea viewing them favorably in 2022 [4]. In 2000, there were only 49 new human immunodeficiency virus (HIV)/acquired immunodeficiency syndrome (AIDS) patients in their 20s and 88 new patients in their 30s; however, in 2020, there were 150 new HIV/AIDS patients in their 20s and 208 new patients in their 30s. In 2020, 295 cases were registered among those in their 20s and 219 cases among those in their 30s, indicating an increasing trend [5].

Safe sexual behaviors refer to preventive sexual behaviors that can protect young adults from sexually transmitted infections resulting from risky sexual behaviors, including not using condoms during intercourse, having oral or anal sex, and having multiple sex partners [2]. Some predictors that influence safe sexual behaviors have been identified in previous studies [6-11].

Sexual body image perception is another factor associated with sexual behaviors. A high tendency regarding risky sexual behaviors was observed among men with a positive body image, while women with a positive body image tended to practice safer sexual behaviors [6].

Sexual role perception is also a factor associated with safe sexual behaviors. According to a study in the United States, female university students with more conventional perceptions about the role of men were less likely to use condoms, whereas female students who had more conventional perceptions about the role of women were more likely to use condoms [7].

Previous studies have found inconsistent associations between sexual attitudes and safe sexual behaviors. In the United States, Boone and Lefkowitz [8] found liberal sexual attitudes to be associated with risky sexual behaviors while drinking alcohol and not using a condom during intercourse with a sex partner. Conversely, in a Taiwanese study, the researchers found no association between sexual attitudes and safe sexual behaviors [9].

Sexual socialization refers to the attitudes of parents and peers toward individuals’ sexual behaviors that can influence one’s safe sexual behaviors [12,13]. According to a qualitative study of Latina mothers, sexual socialization through appropriate sex education by parents could promote safe sexual behaviors in their children [10].

Sexual communication between individuals and their parents or colleagues could also substantially influence safe sexual behaviors. A study conducted in the United States on sexually active university students found that when young adults had more sexual communication, they tended to report safer sexual behaviors. Self-efficacy regarding sexual communication was positively associated with actual sexual communication [11].

The Theory of Planned Behavior (TPB) developed by Ajzen explains how individuals decide to behave and what factors influence their intention to adopt certain health behaviors [14,15]. Three factors influence one’s intentions regarding health behaviors: attitudes, subjective norms, and perceived behavioral control [14,15]. Attitudes refer to one’s personal disposition toward a health behavior and to an individual’s specific evaluation, whether favorable or not, toward that health behavior. Subjective norms refer to the subjective feeling of pressure from society regarding whether to perform certain health behaviors. This influences how we view others’ specific behaviors and our perception of others’ attitudes [16]. Perceived behavioral control refers to an individual’s perception of their ability to control a specific health behavior. This tends to depend on the perception of certain internal factors, such as social resources and support from others [15,16].

Many studies have applied the TPB model to predict the safe sexual behaviors of young adults [17-19-21]. For example, a 2022 study from China used the TPB to identify the association between safe sexual behaviors and sexual knowledge in college students [18]. The group with a higher score for sexual knowledge, attitudes, and safe sexual behavioral intentions showed safer sexual behaviors. In the study, the factors that affected safe sexual behaviors were sexual attitudes, subjective norms, sexual socialization, and perceived behavioral control. In addition, a study from Taiwan on female adolescents (aged 15 to 24 years) also applied the TPB model and confirmed that better sexual communication can affect safe sex behaviors by promoting the intention to use condoms [17].

However, since the TPB mainly focuses on cognitive aspects of human behavior and explains a series of processes about how individuals change their health behaviors, the existing basic TPB model is limited in its ability to identify sufficient evidence regarding the factors associated with sexual behaviors [22]. First, the basic TPB model does not consider various environmental factors such as cultural background or self-body image conception when predicting individuals’ attitudes, subjective norms, and perceived behavioral control. Second, the basic TPB does not account for the possibility of moderating effects or interactions according to specific factors or the characteristics of the target population (such as sex, age, socioeconomic status, and others).

According to a previous study that used the TPB model to identify factors associated with contraception-related behaviors among unmarried young adults in Korea, there are significant differences between men and women in terms of exposure to sexual content and the intention to use contraception. In the study, male participants tended to be influenced directly when exposed to sexual content through various pathways. For the female participants, the intention to use contraception was mediated by their attitudes toward contraception [22]. Another study identified gender differences when predicting safer sexual behaviors among university students in Korea using the TPB model. Communication was found to have a direct effect on female students regarding safer sexual behaviors, while it showed no significant effect on male students [23].

Therefore, given the effect of demographic characteristics and environmental factors on sexual attitudes, subjective norms, and behavioral control, sexual body image and stereotypes regarding gender roles were added as external variables in our study. Furthermore, we specifically examined the direct and indirect effects of these five factors on safe sexual behaviors by expanding the TPB model, which revealed differences between men and women. Based on this context, the hypotheses of this study were as follows.

• Hypothesis 1: Based on the TPB, sexual attitudes, sexual socialization, and sexual communication would be associated with safe sexual behaviors in young adults.

• Hypothesis 2: Sexual body image perception and sexual role perception would act as exogenous variables in the expanded TPB.

• Hypothesis 3: Gender differences exist in the path toward safe sexual behaviors in young adults.

## Methods

**Ethics statement:** This study was performed after obtaining approval from the Institutional Review Board Committee of Kyung Hee University (KHSIRB-21-245). All study participants provided written informed consent.

### Study design

**Figure 1.**
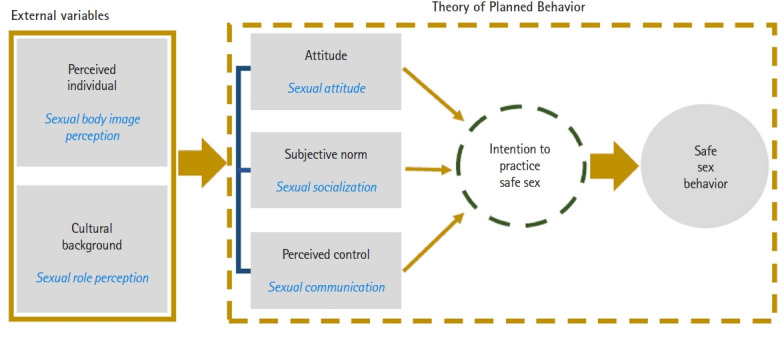
Hypothetical model based on the Theory of Planned Behavior.

This study employed structural equation modeling to identify the factors that affect safe sexual behaviors among Korean men and women of reproductive age. After constructing a theoretical model about safe sexual behaviors based on Ajzen’s TPB model, data were collected from young adults to verify the suitability and hypothesis of the model (Figure 1) [14]. This study followed the STROBE (Strengthening the Reporting of Observational Studies in Epidemiology) reporting guidelines (http://www.strobe-statement.org).

### Participants

Men and women of reproductive age from Korea were recruited based on the following inclusion criteria: (1) unmarried adults in their 20s and 30s who had sex within the last 6 months, (2) whose parents were still alive at the time of data collection (for purposes of the larger study), and (3) who understood the purpose of this study and had no difficulty understanding the questionnaire. The exclusion criteria were as follows: (1) adults who were homosexual or unsure of their sexual orientation, and (2) currently receiving treatment for sexual and reproductive health disorders.

Based on the cohorts included in previous studies [24-26], we aimed to include 200 men and 200 women in this study, and a total of 444 adults (222 men and 222 women) were recruited given a possible dropout rate of 10% [9]. Due to incomplete input and errors in the data collection process, we excluded data from seven participants. Data from 437 respondents were used in the final analysis.

### Measurements

The following instruments were piloted with 40 adults (20 men and 20 women) to verify and improve the applicability of the and to reduce ambiguity due to cultural differences. In this process, two items dealing with asking partners about taking drugs via intravenous injection just before sex and anal sex, both of which are uncommon in Korean culture, were deleted. A total of 85 items were ultimately used in this study. The original version of all scales were translated and back-translated by a bilingual native speaker, and the items were modified for the Korean cultural context. Next, content validity index was evaluated by nursing professors who had experience conducting similar research, and the content validity index of all of the items was verified as being .80 or higher.

#### Safe sexual behaviors

Safe sexual behaviors in this study were assessed using the Safe Sex Behavior Questionnaire (SSBQ) developed by DiIorio et al. [27]. This 24-item tool was designed to measure the frequency with which recommended practices were used to reduce the risk of exposure to and transmission of sexually transmitted infections. It contains 15 positive and nine negative items across five sub-factors: risky behaviors, assertiveness, condom use, avoidance of bodily fluids, and avoidance of anal sex. Each item is rated on a 4-point scale from 1 (never) to 4 (always), and a higher total score (possible range, 24–96 points) indicates more frequent adoption of safe sexual behaviors. Cronbach’s α was .88 upon the tool’s development [27] and .80 in this study.

#### Sexual attitudes

Sexual attitudes in this study were measured using the Brief Sexual Attitude Scale (BSAS) developed by Hendrick et al. [28]. This 23-item tool was designed to assess multidimensional attitudes toward sex and consists of four subscales: permissiveness (10 items), birth control (three items), communication (five items), and instrumentality (five items). Each item is rated on a 5-point Likert scale from 0 (strongly agree) to 4 (strongly disagree). The total score is calculated (possible range: 0–92 points), and lower scores indicate greater permissiveness in sex. Cronbach’s α was .89 upon the tool’s development [28] and .88 in this study.

#### Sexual socialization

Sexual socialization was measured by the Sexual Socialization Instrument (SSI) developed by Lottes and Kuriloff [29]. This 20-item tool measures the influence of peers and parents on young adults regarding sexual permissiveness. The SSI consists of two subscales: parental and peer sexual socialization. Each item is rated on a 5-point Likert scale from 1 (strongly agree) to 5 (strongly disagree). A higher total score (possible range; 20–100 points) indicates a more permissive attitude among parents and peers according to the respondents. Cronbach’s α was .90 upon the tool’s development [29] and .79 in this study.

#### Sexual communication

Sexual communication was measured by the Dyadic Sexual Communication Scale (DSCS) developed by Catania [30]. The DSCS is a 13-item scale that measures a subject’s perception of sexual communication with their partners. Items are rated on a 6-point Likert-type scale from 1 (strongly disagree) to 6 (strongly agree). The total possible score ranges from 13 to 78 points. Cronbach’s α was .85 upon the tool’s development [30] and .82 in this study.

#### Sexual body image perception

Perceived individual body image was measured by the Sexual Body Image Worry (SBIW) scale [31]. The SBIW consists of five items that measure body image concerns rated on a 5-point scale from 0 (not concerned at all) to 4 (very concerned), with a higher total score (possible range: 0 to 20 points) indicating greater concern about one’s sexual body image. Cronbach’s α was .87 upon the tool’s development [31] and .87 in this study.

#### Sexual role perception

The Double Standard Scale (DSS) developed by Caron et al. [32] was used to measure beliefs regarding traditional sexual double standards. This 10-item tool uses a 5-point Likert scale ranging from 1 (strongly agree) to 5 (strongly disagree). A lower total score (possible range: 10–50) indicates greater adherence to traditional sexual double standards. Cronbach’s α was .86 upon the tool’s development [32] and .89 in this study.

### Data collection

Data were collected between January 3 and January 28, 2022, using an online survey platform because of the sensitivity of the contents and the coronavirus disease 2019 pandemic. A study recruitment banner was posted on Social Network Service (e.g. instagram, facebook) and potential participants could access the online survey through the link or QR code for voluntary participation. The survey took approximately 20 to 30 minutes for completion and a e-coupon valued at 20 US dollars was provided as compensation.

### Data analysis

Descriptive statistics were used to analyze the general characteristics and measurement variables. The correlation between variables was analyzed using Pearson correlation coefficients. In addition, we used a structural equation model to identify the paths and effects of the variables that affected safe sexual behaviors. All statistical analyses were formed using SPSS and AMOS (version 24.0; IBM Corp., Armonk, NY, USA).

To confirm the construct validity of the measurement variables, exploratory factor analysis was performed to determine whether each item was classified in the same dimension for each measurement variable. Inappropriate items with factor loadings of less than 0.7 were removed. A total of 85 items out of an initial 95 were ultimately used for the final analysis after deleting 10 items: three items from the SSBQ (no. 15 [“I engage in anal intercourse”], no. 16 [“I ask my potential sexual partners about their history of IV drug use”], and no. 23 [“I engage in anal intercourse without using a condom”]), three items from the BSAS (no. 1 [“I do not need to be committed to the person to have sex with him/her”], no. 18 [“Sex is usually an intensive, almost overwhelming experience”], and no. 23 [“Sex is primarily a bodily function, like eating”]), one item from the DSCS (no. 1 [“My partner rarely responds when I want to talk about our sex life”]), and three items from the SSI (no. 6 [“According to my parents, having sexual intercourse is an important part of my becoming an adult”], no. 15 [“My friends suggest dates to each me who are known to be sexually open”], and no. 16 [“My parents encourage me to have sex with people before I get married.”]). The Cronbach’s α values of these measurements ranged from 0.79 to 0.89 in this study (Supplementary Table 1).

The chi-square statistic (degrees of freedom), goodness-of-fit index (GFI), adjusted GFI (AGFI), normed fit index (NFI), Tucker-Lewis index (TLI), comparative fit index (CFI), root mean residual (RMR), and root mean square error of approximation (RMSEA) were used to evaluate the fit for the hypothetical model. A moderation effect analysis was performed to analyze the differences between men and women. The cutoff value for the GFI, AGFI, NFI, TLI, and CFI was 0.90, and the RMR and RMSEA were set at 0.1 [24].

## Results

### Participants’ characteristics and measurement variables

Table 1 shows the general characteristics of the study sample according to gender. The highest proportion of both men (n=116, 64.4%) and women (n=64, 35.6%) were 30-39 years of age. A total of 52 men (51.5%) had a high school education, whereas 169 women (50.3%) had a college-level education or higher. Most of the men were employed (n=156, 50.2%) and resided in a metropolitan area (n=169, 52.2%), while most of the women were students (n=63, 50%) and resided in non-metropolitan areas (n=63, 50%).

The mean scores (and standard deviations) for each variable were as follows. The BSAS score was statistically significantly higher among men (13.43±4.18) than among women (10.35±2.83), as was the SSI score (men, 41.92±8.16; women, 38.63±7.77). The mean DSCS score between men (48.56±5.79) and women (46.16±5.43) was similar, as was the mean SBIW score (men, 16.23±4.51; women, 15.09±4.21). The mean DSS score was statistically significantly higher in men (34.84±6.04) than women (30.68±5.97), whereas the SSBQ score was higher among women (53.25±7.93) than among men (49.24±8.56) (Table 1).

### Evaluation of normality and multicollinearity

Each variable was tested for normality and multicollinearity. The absolute value of skewness and kurtosis of all variables in both men and women groups did not exceed the critical value (1.96) at a significance level of 0.05, which means that the sample satisfied the assumption of normality. In order to test multicollinearity between measurement variables, the correlation coefficients between variables were calculated (Table 2). The correlation coefficients of the study variables ranged from 0.01 to 0.34, which indicates that there was no multicollinearity between the measured variables.

### Evaluation of hypothetical models

According to the overall model fit of the hypothetical model, the *χ*^2^ (137.43; degrees of freedom=5, *p*<.001), GFI (0.90), AGFI (0.87), NFI (0.85), TLI (0.87), and CFI (0.89) values were acceptable, while the RMR (0.08) and RMSEA (0.06) values were borderline acceptable.

### Direct, indirect, and total effects of variables

**Figure 2.**
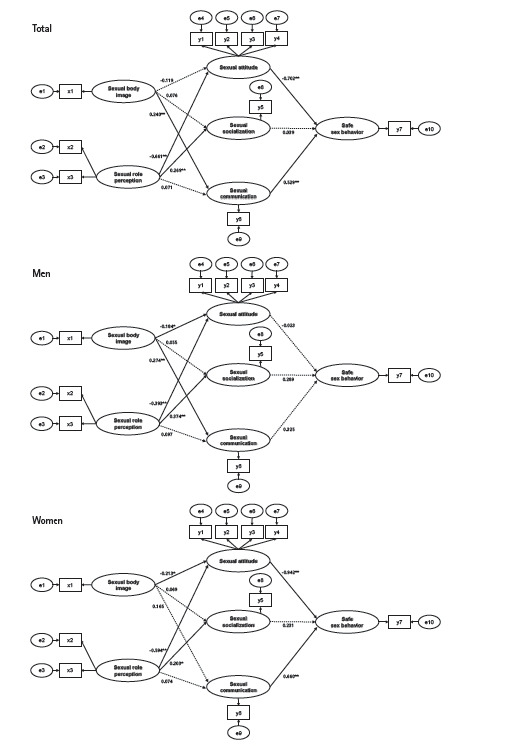
Path diagrams of the final models for men and women.

In the overall model, five out of nine paths were found to be statistically significant (Figure 2). There was a direct effect between sexual role perception and sexual attitudes (β=–.66, *p*<.001). Sexual role perception also had a direct effect on sexual socialization (β=.27, *p*<.001). Sexual body image had a direct effect on sexual communication (β=.24, *p*<.001). The variables that had a direct effect on safe sexual behaviors were sexual attitudes (β=–.70, *p*<.001) and communication (β=.53, *p*<.001), while an indirect effect was found for sex role perception (β=.42, *p*<.001), explaining 49.4% of variance in safe sexual behaviors (Table 3).

### Comparison of paths according to sex

Figure 2 and Table 4 show the overall path toward safe sexual behaviors and gender differences. Three paths showed the same level of significance for both men and women (*p*<.05). The paths between sexual body image and sexual attitudes were significant in both men and women models (men, β=–.18; women, β=–.21). Sexual role perception had a significant effect on sexual attitudes (men, β=–.39; women, β=–.59) and sexual socialization (men, β=.37; women, β=.20).

In men, however, sexual body image had a significant effect on sexual communication (β=.27, *p*<.001), whereas in women, it had no significant effect. Additionally, for women, sexual attitudes (β=–.94, *p*<.001) and communication (β=.66, *p*<.001) had a significant effect on safe sexual behaviors, but these factors had no effect on men.

## Discussion

Our study confirmed that sexual attitudes and communication directly affect safe sexual behaviors and that the perception of sexual roles indirectly affects safe sexual behaviors in young adults, explaining 49% of the safe sex model.

A study conducted in China reported that sexual attitudes had a mediating effect on knowledge of risky sex and sexual behaviors among university students [33]. Prior studies reported that individuals with negative sexual attitudes were less likely to use condoms during sex than those with positive sexual attitudes [7] and having a positive attitude toward implementing safe sexual behaviors was associated with actual safe sexual behaviors in women with economic decision-making autonomy [34]. However, Yang et al. [35] reported that an open attitude toward sexual partners tended to correspond to an open attitude toward premarital sex and homosexuality, which can increase the likelihood of adopting risky sexual behaviors. Furthermore, multiple previous studies reported no significant correlation between positive sexual attitudes and safe sexual behaviors [8,9,21]. Thus, this study provides empirical evidence that a more permissive attitude toward sex leads to safer sexual behaviors.

This study also demonstrated that sexual communication has a significant effect on safe sexual behaviors. These findings are consistent with those of previous studies of young adults [17], adolescents [36], and high school students [37] that found condom use to be more frequent among respondents who discussed sexual intercourse with their partners. In a previous meta-analysis and study, more communication between adolescents and their parents about sex had a significant effect on the use of condoms and other contraceptives [13,38]. Gause et al. [39] also found that infrequent sexual communication with a sexual partner regarding contraception and sexually transmitted infections was significantly associated with a lower probability of condom use in female adolescents. A meta-analysis that examined the relationship between sexual communication between partners and condom use also confirmed that sexual communication is an important factor in determining safe sexual behaviors [11].

This study also showed that the perception of sexual roles has a significant effect on sexual attitudes and communication. In other words, a lower degree of prejudice regarding sexual roles corresponded to a higher degree of generosity regarding the sexuality of one’s partner and more frequent communication with one’s sexual partners. This result is consistent with a study that observed differences in attitudes toward premarital intercourse and the perception of dyadic sexual roles by gender [40]. There were significant associations between sex role perception and sexual attitudes. Furthermore, female subjects with more traditional expectations regarding sexual roles were more likely to have a double standard regarding premarital sexual relationships [40]. In terms of the relationship between sexual role perception and communication with partners, our study was also consistent with a previous study from the United States [7] that reported that those with a less traditional perception of sexual roles were more likely to talk with their partners about sexual issues, and this kind of interaction made them more satisfied with their relationships.

This study also found that a better perception of one’s sexual body image corresponded to better sexual communication with one’s partner. This result is consistent with those of previous studies that found that a more positive self-body image corresponded to more positive determinants of sexual communication [41,42]. It also corresponds to the results of a past study that found a positive body image to be associated with better communication about sex with partners [43].

In this study, sexual socialization did not have a significant effect on safe sexual behaviors. According to a qualitative study of Latina mothers, sexual socialization through appropriate sex education by parents can promote safe sexual behaviors in their children [10]. One study from the United States observed no associations between communication with parents and condom use in adolescents [44]. Another controversial study observed that peer norms and peer socialization were marginally significant predictors of condom use among college students [8]. These differences between studies may be a result of differences in the ages of the study participants, which can affect the level of influence of peers and parents according to the subjects’ psychosocial development. Thus, further studies are needed to clarify the interplay between sexual socialization and safe sexual behaviors.

This study confirmed that there were differences between men and women in terms of factors that affect safe sexual behaviors. Men with a positive body image tended to be more comfortable communicating with their sexual partners whereas women with a positive body image were less comfortable, which is inconsistent with a recent study that reported that sexual self-esteem acted as a serial mediator in the relationship between sexual body image and sexual communication in Chinese women [45]. The authors of the previous study also emphasized that sexual communication with a partner can play a unique mediating role in building women’s sexual body image through sexual functions such as arousal, orgasm, and lubrication. However, a study from Australia revealed that, among young and older adults, no association was observed between sexual body image and psychological interaction with one’s sexual partners in both men and women [46]. The conflicting results of these preceding studies suggest that there may be differences between Eastern and Western cultures in how sexual body image is interpreted. Since sexual behaviors are influenced by various factors, including gender, ethnicity, and cultural factors [47], further exploratory research in this area is recommended.

This study’s finding that sexual attitudes and communication were significant variables affecting the safe sexual behaviors of women but not in men aligns with those of multiple previous studies [48-51]. For example, a study from China showed a significant gap in sexual attitudes; in particular, male students were more likely to agree to premarital sex and one-night stands and have more than one sex partner, whereas young female students had a greater intention to use condoms [48]. Another study conducted in Europe also identified gender differences in condom use. Spanish and Portuguese female college students had more positive attitudes toward condom use than male college students and were more confident in communicating with their partners about condom use during sex as a measure for preventing HIV [50]. Another study of university students in the Mid-Atlantic United States found that female students had more positive sexual attitudes than male students [51].

The paths between sexual body image and sexual attitudes were significant in men and women models, respectively, compared to the overall model. This result is similar to that of a study from the United States that found that women with a more positive body image had more permissive and confident sexual attitudes [52]. Another study from the United Kingdom showed similar results among male and female undergraduate students, in which students with a more negative body image were more likely to have avoidant and hesitant sexual attitudes [53]. However, Gillen et al. [6] found that male individuals with a more positive body image were more likely to have sexual double standards. Overall, these findings indicate the substantial influence of sexual body image on the sexual attitudes of both men and women.

This study yielded further insights into safe sexual behaviors among young adults by constructing an extended TPB model that reflected the participants’ characteristics and cultural factors, leading to increased explanatory power. Another strength is that it confirmed the existence of gender differences in the factors that explain safe sexual behaviors. Nevertheless, the findings are still insufficient for explaining the causal relationships among these variables due to the study’s cross-sectional design. In future studies, a longitudinal design may help address this limitation.

In this study, we confirmed the existence of gender differences in predictors of safe sexual behaviors among Korean adults in their 20s to 30s. Men participants were more likely to be indirectly influenced by their perceived body image in the path toward sexual communication with their partners and safe sexual behaviors. Women participants, however, were more likely to be affected by interpersonal or social factors than men participants. Women participants were directly influenced by sexual attitudes and sexual communication formed in social relationships when undertaking safe sexual behaviors. Therefore, gender differences should be considered when examining factors that affect safe sexual behaviors. In addition, since students are exposed to sexual content through mass media, sex education should be provided that includes proper condom usage, a positive attitude toward contraception, and sufficient sexual communication between partners.

## Figures and Tables

**Table 1.** Characteristics of participants (N=437)

Variable	Categories	n (%) or mean±SD			*χ*^2^/t	*p*
		Total (n=437)	Men (n=219)	Women (n=218)		
Age (year)	20-29	257 (58.5)	103 (40.1)	154 (59.9)	24.74	<.001
	30-39	180 (41.2)	116 (64.4)	64 (35.6)		
Education level	<High school	101 (23.1)	52 (51.5)	49 (48.5)	0.17	.678
	≥College	336 (76.9)	167 (49.7)	169 (50.3)		
Occupation	Worker	311 (71.2)	156 (50.2)	155 (49.8)	0.02	.893
	Student	126 (28.8)	63 (50.0)	63 (50.0)		
Area of residence	Metropolitan	324 (74.1)	169 (52.2)	155 (47.8)	1.69	.194
	Non-metropolitan	113 (25.9)	50 (44.5)	63 (55.5)		
	Possible score range					
Safe sexual behaviors	24–96	51.69 ± 8.61	49.24±8.56	53.25±7.93	–3.84	<.001
Sexual attitudes	0–92	11.89 ± 4.11	13.43±4.18	10.35±2.83	7.31	<.001
Sexual socialization	20–100	40.54 ± 8.03	41.92±8.16	38.63±7.77	4.34	<.001
Sexual communication	13–78	47.23 ± 5.77	48.56±5.79	46.16±5.43	2.62	.009
Sexual body image perception	0–20	15.91± 4.50	16.23±4.51	15.09±4.21	2.74	.006
Sexual role perception	10–50	32.16 ± 6.01	34.84±6.04	30.68±5.97	7.30	<.001

**Table 2.** Correlations between safe sexual behaviors, sexual attitudes, sexual socialization, sexual communication, sexual body image perception, and sexual role perception (N=437)

Variable	r (*p*)				
	Safe sexual behaviors	Sexual attitudes	Sexual socialization	Sexual communication	Sexual body image
Safe sexual behaviors	1				
Sexual attitudes	.21 (<.001)	1			
Sexual socialization	.10 (.047)	.17 (<.001)	1		
Sexual communication	.15 (<.001)	–.34 (<.001)	–.19 (<.001)	1	
Sexual body image	–.10 (.046)	.01 (.884)	.08 (.082)	–.18 (<.001)	1
Sexual role perception	–.25 (<.001)	–.29 (<.001)	–.08 (.117)	–.05 (.317)	.29 (<.001)

**Table 3.** Direct, indirect, and total effects in the model (N=437)

Endogenous variable	Exogenous variables	SMC	β (*p*)		
			Direct effect	Indirect effect	Total effect
Sexual attitudes	Sexual body image	0.23	–.12 (.051)	-	–.12 (.051)
	Sexual role perception		–.66 (<.001)	-	–.66 (<.001)
Sexual socialization	Sexual body image	0.25	.08 (.150)	-	.08 (.150)
	Sexual role perception		.27 (<.001)	-	.27 (<.001)
Sexual communication	Sexual body image	0.37	.24 (<.001)	-	.24 (<.001)
	Sexual role perception		.07 (.190)	-	.07 (.190)
Safe sexual behaviors	Sexual body image	0.49	-	.05 (.110)	.05 (.110)
	Sexual role perception		-	.42 (<.001)	.42 (<.001)
	Sexual attitudes		–.70 (<.001)	-	–.70 (<.001)
	Sexual socialization		.04 (.640)	-	.04 (.640)
	Sexual communication		.53 (<.001)	-	.53 (<.001)

SMC, Squared multiple correlation; β, standardized coefficient.

**Table 4.** Comparison of paths according to gender (N=437)

Exogenous variables	Endogenous variables	Men		Women	
		Standardized coefficient	CR (*p*)	Standardized coefficient	CR (*p*)
Sexual body image	Sexual attitudes	–.18	–2.39 (.017)	–.21	–2.11 (.035)
Sexual role perception	Sexual attitudes	–.39	–4.79 (<.001)	–.59	–4.29 (<.001)
Sexual body image	Sexual socialization	.06	0.79 (.428)	.07	0.88 (.378)
Sexual role perception	Sexual socialization	.37	5.35 (<.001)	.20	2.69 (.007)
Sexual body image	Sexual communication	.27	3.52 (<.001)	.17	1.96 (.050)
Sexual role perception	Sexual communication	.10	1.31 (.191)	.07	0.93 (.353)
Sexual attitudes	Safe sexual behaviors	–.02	–0.19 (.852)	–.94	–3.55 (<.001)
Sexual socialization	Safe sexual behaviors	.29	1.88 (.061)	.23	1.60 (.109)
Sexual communication	Safe sexual behaviors	.33	1.73 (.084)	.66	3.81 (<.001)

CR, Critical ratio.

## Supplementary materials

Further details on supplementary materials are presented online (available at https://doi.org/10.4069/kjwhn.2023.06.16).

Supplementary Table 1. Final numbers of items and Cronbach’s α of instruments

## References

[1] World Health Organization (2022). Sexually transmitted infections (STIs) [Internet]. https://www.who.int/news-room/fact-sheets/detail/sexually-transmitted-infections-(stis).

[2] Centers for Disease Control and Prevention (2022). Sexually transmitted disease surveillance 2020 [Internet]. https://stacks.cdc.gov/view/cdc/125947.

[3] Holway GV, Brewster KL, Tillman KH (2020). Condom use at first vaginal intercourse among adolescents and young adults in the United States, 2002-2017. J Adolesc Health.

[4] Korean Statistical Information Service (2022). Korean Social Trends 2022 [Internet]. https://sri.kostat.go.kr/menu.es?mid=a90104010100.

[5] Korean Disease Control and Prevention Agency (2021). Annual report on the notified HIV/AIDS in Korea, 2021 [Internet]. https://npt.kdca.go.kr/npt/biz/npp/portal/nppPblctDtaView.do?pblctDtaSeAt=1&pblctDtaSn=2645.

[6] Gillen MM, Lefkowitz ES, Shearer CL (2006). Does body image play a role in risky sexual behavior and attitudes?. J Youth Adolesc.

[7] Lefkowitz ES, Shearer CL, Gillen MM, Espinosa-Hernandez G (2014). How gendered attitudes relate to women’s and men’s sexual behaviors and beliefs. Sex Cult.

[8] Boone TL, Lefkowitz ES (2004). Safer sex and the health belief model: considering the contributions of peer norms and socialization factors. J Psychol Human Sex.

[9] Lou JH, Chen SH (2009). Relationships among sexual knowledge, sexual attitudes, and safe sex behaviour among adolescents: a structural equation model. Int J Nurs Stud.

[10] Leija SG (2022). Sexual socialization: a qualitative exploration of immigrant Latina mothers’ perception of sex-communication with their adolescent daughters.

[11] Li Y, Samp JA (2017). Sexual relationship power, safer sexual communication, and condom use: a comparison of heterosexual young men and women. West J Commun.

[12] Flores D, Barroso J (2017). 21st century parent-child sex communication in the United States: a process review. J Sex Res.

[13] Widman L, Choukas-Bradley S, Noar SM, Nesi J, Garrett K (2016). Parent-adolescent sexual communication and adolescent safer sex behavior: a meta-analysis. JAMA Pediatr.

[14] Kuhl J, Beckmann J, Ajzen I, Kruglanski AW, Klar Y, Gollwitzer PM (1985). Action control form cognition to behavior.

[15] Ajzen I (1991). The theory of planned behavior. Organ Behav Hum Decis Process.

[16] Brookes E (2021). The theory of planned behavior: behavioral intention [Internet]. Simply Psychology official portal. https://www.simplypsychology.org/theory-of-planned-behavior.html.

[17] Tseng YH, Cheng CP, Kuo SH, Hou WL, Chan TF, Chou FH (2020). Safe sexual behaviors intention among female youth: the construction on extended theory of planned behavior. J Adv Nurs.

[18] Wang X, Jin Y, Tian M, Zhuo Q, Lin CL, Hu P (2022). Safe-sex behavioral intention of Chinese college students: examining the effect of sexual knowledge using the theory of planned behavior. Front Psychol.

[19] Moeini B, Hazavehei SM, Zareban I, Mousali A, Bashiriyan S, Soltanian AR (2017). Effectiveness of an educational program based on the theory of planned behavior for improving safe sexual behaviors intention among addicted males: a quasi experimental study. Int J High Risk Behav Addict.

[20] Je M, Ju HO, Lee J (2020). Factors affecting reproductive health promotion behavior among late-adolescent girls in South Korea: a cross-sectional descriptive study. Child Youth Serv Rev.

[21] Lin CL, Ye Y, Lin P, Lai XL, Jin YQ, Wang X (2021). Safe sexual behavior intentions among college students: the construction of an extended theory of planned behavior. Int J Environ Res Public Health.

[22] Hwang SW, Chung CW (2014). Structural equation modeling on contraception behavior of unmarried men and women in Korea: gender difference. J Korean Acad Nurs.

[23] Kim YJ (2014). A predictive model for safer sexual behavior among Korean university students: based on the theory of planned behavior [dissertation].

[24] Yu JP (2012). The concept and understanding of structural equation modeling.

[25] Hair JF, Black WC, Babin BJ, Anderson RE, Tatham RL (2006). Multivariate data analysis.

[26] DeVellis RF (2017). Scale developemnt: theory and application.

[27] DiIorio C, Parsons M, Lehr S, Adame D, Carlone J (1992). Measurement of safe sex behavior in adolescents and young adults. Nurs Res.

[28] Hendrick C, Hendrick SS, Reich DA (2006). The brief sexual attitudes scale. J Sex Res.

[29] Lottes IL, Kuriloff PJ (1994). Sexual socialization differences by gender, Greek membership, ethnicity, and religious background. Psychol Women Q.

[30] Catania JA (1987). Help-seeking: an avenue for adult sexual development.

[31] Bauer GR, Hammond R (2015). Toward a broader conceptualization of trans women’s sexual health. Can J Hum Sex.

[32] Caron SL, Davis CM, Halteman WA, Stickle M (1993). Predictors of condom‐related behaviors among first‐year college students. J Sex Res.

[33] Guan M (2021). Sexual and reproductive health knowledge, sexual attitudes, and sexual behaviour of university students: findings of a Beijing-based survey in 2010-2011. Arch Public Health.

[34] Imo CK, Odimegwu CO, De Wet-Billings N (2022). Women’s attitudes towards negotiating safe sexual practices in Nigeria: do family structure and decision-making autonomy play a role?. BMC Womens Health.

[35] Yang XH, Yuan S, Zhang R, Yu JF, Nzala SH, Wang PG (2019). Risky sexual behaviors and associated factors among college students in Lusaka, Zambia. Arch Sex Behav.

[36] Widman L, Noar SM, Choukas-Bradley S, Francis DB (2014). Adolescent sexual health communication and condom use: a meta-analysis. Health Psychol.

[37] Namisi FS, Aarø LE, Kaaya S, Onya HE, Wubs A, Mathews C (2013). Condom use and sexuality communication with adults: a study among high school students in South Africa and Tanzania. BMC Public Health.

[38] Rogers AA (2016). Parent–adolescent sexual communication and adolescents’ sexual behaviors: a conceptual model and systematic review. Adolesc Res Rev.

[39] Gause NK, Brown JL, Welge J, Northern N (2018). Meta-analyses of HIV prevention interventions targeting improved partner communication: effects on partner communication and condom use frequency outcomes. J Behav Med.

[40] Zuo X, Lou C, Gao E, Cheng Y, Niu H, Zabin LS (2012). Gender differences in adolescent premarital sexual permissiveness in three Asian cities: effects of gender-role attitudes. J Adolesc Health.

[41] Gillen MM, Markey CH (2019). A review of research linking body image and sexual well-being. Body Image.

[42] Gillen MM, Markey CN (2014). Body image and HIV risk among college students. Am J Health Behav.

[43] Ramseyer Winter V, Gillen MM, Kennedy AK (2018). Associations between body appreciation and comfort communicating about sex: a brief report. Health Commun.

[44] Wright PJ, Herbenick D, Paul B (2020). Adolescent condom use, parent-adolescent sexual health communication, and pornography: findings from a U.S. probability sample. Health Commun.

[45] Wu T, Zheng Y (2021). Effect of sexual esteem and sexual communication on the relationship between body image and sexual function in Chinese heterosexual women. J Sex Med.

[46] Davison TE, McCabe MP (2005). Relationships between men’s and women’s body image and their psychological, social, and sexual functioning. Sex Roles.

[47] Ahrold TK, Meston CM (2010). Ethnic differences in sexual attitudes of U.S. college students: gender, acculturation, and religiosity factors. Arch Sex Behav.

[48] Lyu J, Shen X, Hesketh T (2020). Sexual Knowledge, Attitudes and behaviours among undergraduate students in China-implications for sex education. Int J Environ Res Public Health.

[49] Valente AM, Auerswald CL (2013). Gender differences in sexual risk and sexually transmitted infections correlate with gender differences in social networks among San Francisco homeless youth. J Adolesc Health.

[50] Muñoz-Silva A, Sánchez-García M, Martins A, Cristina N (2009). Gender differences in HIV-related sexual behavior among college students from Spain and Portugal. Span J Psychol.

[51] Martin JC, Mak JY (2013). College students’ sexual knowledge and attitudes. KAHPERD J.

[52] Ackard DM, Kearney-Cooke A, Peterson CB (2000). Effect of body image and self-image on women's sexual behaviors. Int J Eat Disord.

[53] La Rocque CL, Cioe J (2011). An evaluation of the relationship between body image and sexual avoidance. J Sex Res.

