# Supplementary material for: Factors affecting the safe sexual behaviors of Korean young adults by gender: a structural equation model
Source: Korean J Women Health Nurs. 2023 Jun 30;29(2):115–27. doi: 10.4069/kjwhn.2023.06.16 (PMC10326550; doi:10.4069/kjwhn.2023.06.16)
Supplement: Supplementary Table 1. — Final numbers of items and Cronbach’s α of instruments [file kjwhn-2023-06-16-Supplementary-Table-1.pdf]

Supplementary Table 1. Final numbers of items and Cronbach's α of instruments

Instrument	Number of items	Deleted items after EFA	Number of final items	Cronbach's α
SSBQ	24	3	21	0.80
BSAS	23	3	20	0.88
DSS	10	0	10	0.89
DSCS	13	1	12	0.82
SBIW	5	0	5	0.87
SSI	20	3	17	0.79
Total items	95	10	85	

BSAS: Brief Sexual Attitude Scale; DSCS: Dyadic Sexual Communication Scale; DSS, Double Standard Scale; EFA: Exploratory Factor Analysis; SBIW: Sexual Body Image Worry; SSBQ: Safe Sex Behavior Questionnaire; SSI: Sexual Socialization Instrument.
